# Differences in tissue-associated bacteria between metastatic and non-metastatic colorectal cancer

**DOI:** 10.3389/fmicb.2023.1133607

**Published:** 2023-06-09

**Authors:** Peng Zhou, Ze Dai, Yaoyao Xie, Tong Li, Zhizheng Xu, Yanhong Huang, Desen Sun, Yuping Zhou

**Affiliations:** ^1^Department of Gastroenterology, The First Affiliated Hospital of Ningbo University, Ningbo, Zhejiang, China; ^2^Institute of Digestive Disease of Ningbo University, Ningbo, China; ^3^Zhejiang Key Laboratory of Pathophysiology, Department of Biochemistry and Molecular Biology, Medical School of Ningbo University, Ningbo, Zhejiang, China; ^4^Department of Colorectal and Anal Surgery, The First Affiliated Hospital of Ningbo University, Ningbo, Zhejiang, China

**Keywords:** colorectal cancer (CRC), metastasis, tissue-associated bacteria, *Bacteroides*, *Streptococcus*

## Abstract

**Background and aims:**

Accumulated evidence indicates that the intestinal microbiota plays crucial roles in the initiation and progression of colorectal cancer (CRC). However, the effects of the tissue-associated microbiota on CRC metastasis are poorly defined. The aim of this study was to explore the differences in bacteria between metastatic and non-metastatic CRC tissues and identify potential bacterial species that associate with CRC metastasis.

**Methods:**

16S rDNA amplicon high-throughput sequencing was used to test the intestinal tissue-associated microbiota in patients with metastatic CRC (*n* = 48) and non-metastatic CRC (*n* = 44). The microbial diversity and differential species were analysed by standard microbiological methods, and then the differential bacteria were confirmed by qPCR. Receiver operating characteristic (ROC) curves were plotted to evaluate the ability of the differential bacteria in predicting the metastasis of CRC. In addition, the microbial compositions of tumor-adjacent tissues from the metastatic and non-metastatic CRC groups were analysed.

**Results:**

The α- or β-diversity of microbial community between the metastatic and non-metastatic CRC groups did not exhibit significant differences. However, some bacterial abundances between two groups showed significant differences. At the phylum level, Bacteroidota and Desulfobacterota were significantly higher in the metastatic group than in the non-metastatic group, while Proteobacteria was significantly decreased in the metastatic group. At the genus level, *Bacteroides* (mainly composed of *Bacteroides fragilis* and *Bacteroides uniformi*s) was significantly higher in the metastatic group than in the non-metastatic group, while *Streptococcus* and *Escherichia-Shigella* were significantly decreased. The ROC curves of the selected bacteria showed area under the curve (AUC) values ranging from 0.598 to 0.69; when CEA and the selected bacteria were combined, the AUC values increased from 0.678 to 0.705. In addition, the bacterial composition of tumor-adjacent tissues from the metastatic and non-metastatic CRC groups were also different, and the differential bacteria were consistent with those between metastatic and non-metastatic CRC tumor tissues.

**Conclusion:**

The bacterial composition of tumor and tumor adjacent tissue from the metastatic CRC group was different from that of the non-metastatic CRC group; in particular, *Bacteroides* was increased, and *Streptococcus* was decreased. These findings are helpful to further reveal the mechanism of CRC metastasis and provide new ideas for the clinical diagnosis and treatment of CRC metastasis.

## Introduction

Colorectal cancer (CRC) is one of the most common gastroenterological tumors. According to 2020 epidemiological data, CRC is the third most common diagnosed and second most deadly cancer worldwide (Sung et al., 2021). The process of intestinal cancer development usually takes 10–15 years, including the initiation, promotion, progression, and metastasis stages (Dekker et al., 2019). Metastasis is known as the main cause of death in CRC patients, with a 5 years survival rate of less than 20% (Pretzsch et al., 2019; Biller and Schrag, 2021). Therefore, it is of great importance and necessity to understand the potential risk factors that promote CRC metastasis, which can serve as targets to block CRC metastasis (Fong et al., 2020).

The pathogenesis of CRC is highly complex and involves both genetic and environmental factors (Biller and Schrag, 2021). In recent years, numerous studies have supported the notion that the intestinal microbiota plays a crucial role in the initiation and progression of CRC (Wong and Yu, 2019; Cheng et al., 2020; Rebersek, 2021). Usually, the microbiota significantly changed in CRC patients, with tumor-promoting bacteria enriched and tumor-inhibiting bacteria depleted. Some bacteria, such as colibactin-producing *Escherichia coli*, enterotoxigenic *Bacteroides fragilis*, and *Fusobacterium nucleatum*, are enriched in the intestinal microbiota (Cheng et al., 2020; Tabowei et al., 2022). They can promote CRC initiation and progression by inducing host DNA damage, stimulating oncogenic pathways related to cell growth and proliferation, or creating a proinflammatory environment (Clay et al., 2022). However, studies focusing on specific bacteria that can promote CRC metastasis are limited.

It has been reported that the bacterial burden in CRC mucosal tissue is higher than that in healthy controls. Interestingly, the composition of the intestinal microbiota and tissue-associated bacteria are significantly different (Keku et al., 2015; Flemer et al., 2017), suggesting that they may play different roles in CRC progression. To date, many studies have revealed the functions of the intestinal microbiota (fecal sample) in CRC, but only a few have focused on tissue-associated bacteria (CRC tissue samples) (Costa et al., 2022). Thus, the features and functions of tissue-associated bacteria in CRC progression and metastasis remain elusive.

In this study, we compared the differences in the tissue-associated microbiota between tumor tissues from metastatic and non-metastatic CRC patients and analysed the clinical value of differential bacteria in the prognosis of CRC metastasis. Our findings will shed light on fully revealing the characteristics of tissue-associated bacteria and provide an effective foundation for the in-depth study of their role in CRC metastasis.

## Materials and methods

### Subjects

We selected patients who were surgically treated for colorectal cancer between January 2020 and December 2021. According to the UICC/AJCC TNM Staging System for CRC (8th edition, 2017), 92 patients with CRC were divided into a metastatic group (*n* = 48) and a non-metastatic group (*n* = 44) by specialist physicians. Exclusion criteria were anal canal tumors, appendiceal tumors, neuroendocrine tumors, familial adenomatous polyposis and cases of additional surgery for perforation or bleeding complicated by endoscopic treatment, colorectal cancer with antibiotics, glucocorticoids or immunosuppressive drugs used within 1 month at the time of sampling, and other colorectal cancer patients who could not be entered into this cohort.

Clinical samples were collected including tumor and tumor-adjacent tissues from CRC patients (the area surrounding the tumor <3 cm was considered adjacent tissue).

The collection of relevant clinical parameters of CRC patients included general clinical information, routine blood tests, biochemistry, coagulation function, tumor markers, histopathology, and other indicators.

### Bacterial DNA extraction

Tissue-associated bacterial DNA was extracted from samples by using the QIAamp DNA Microbiome Kit (Qiagen, Hilden, Germany) according to the manufacturer’s protocol. In brief, approximately 100 mg of intestinal tissue was homogenized, host cells were lysed, and host DNA was digested by benzonase (human DNase) while leaving the bacterial cells intact. Then, bacterial cells were concentrated by centrifugation, and bacterial DNA was extracted.

### 16S rDNA amplicon sequencing and analysis

The sequencing procedure was performed as previously described (Emery et al., 2020). Briefly, the V3–V4 hypervariable region of bacterial 16S rDNA was amplified using universal sequencing primers 341F 5′-CCTAYGGGRBGCASCAG-3′ and 806R 5′-GGACTACNNGGGTATCTAAT-3′ (Yuan et al., 2018). The amplicon was sequenced by the Illumina MiSeq PE300 platform at Majorbio Bio-Pharm Technology Co., Ltd. (Shanghai, China). Raw FASTQ files were de-multiplexed and quality-filtered by QIIME1 (V1.9.1).^1^ The optimized sequences were clustered into operational taxonomic units (OTUs) using UPARSE 7.1^2^ with 97% sequence similarity level. The most abundant sequence for each OTU was selected as a representative sequence. To minimize the effects of sequencing depth on alpha- and beta-diversity measure, the number of 16S rRNA gene sequences from each sample were rarefied to 20,000. Bioinformatic analysis of the microbiota was based on the OTUs information. Alpha diversity indices including Chao1 richness and Shannon index were calculated with Mothur v1.30.1 (Emery et al., 2020). The similarity among the microbial communities in different samples was determined by non-metric multidimensional scaling analysis (NMDS) based on Bray-curtis dissimilarity using Vegan v2.5-3 package. The linear discriminant analysis (LDA) effect size (LEfSe) (Segata et al., 2011)^3^ was performed to identify the significantly abundant taxa (phylum to genera) of bacteria among the different groups (LDA score > 3, *P* < 0.05).

### Real-time quantitative PCR (qPCR) analysis

The main differential bacteria and *Bacteroides fragilis* toxin gene were confirmed by qPCR analysis. Briefly, experiments were performed with a QuantStudio 3 Real-time PCR System (Thermo Fisher Scientific, USA). The qPCR reaction system was: 2 × SYBR Green premix [Takara Bio technology (Beijing) Co., Ltd. Beijing, China] 5 μL, 1 μM forward and reverse primer sets (Table 1) 2 μL, 20 ng/μL DNA template 1 μL, ddH_2_O 2 μL. The conditions of qPCR reaction were as follows: initial denaturation was done at 95°C for 60 s; amplification by using 45 cycles including denaturation at 95°C for 5 s, annealing and extension at 60°C for 30 s; melting curve was done at 95°C for 15 s, 60°C for 60 s; 95°C for 30 s.

**TABLE 1:** Quantitative polymerase chain reaction (qPCR) primers for target bacteria and *Bacteroides fragilis* toxin gene.

Bacteria	Primer sequences (5’–3’)	Product size (bp)	References
Bacteroidota	CATGTGGTTTAATTCGATGAT	126	Queipo-Ortuño et al., 2012
	AGCTGACGACAACCATGCAG		
Proteobacteria	CATGACGTTACCCGCAGAAGAAG	195	Queipo-Ortuño et al., 2012
	CTCTACGAGACTCAAGCTTGC		
*Bacteroides*	GGTTCTGAGAGGAGGTCCC	106	Queipo-Ortuño et al., 2012
	GCTGCCTCCCGTAGGAGT		
*Streptococcus*	ACGGTCTTGCTGTCACTTATA	257	Johnson et al., 2016
	TACACATATGTTCTTCCCTAATAA		
*Escherichia-Shigella*	GAGTAAAGTTAATACCTTTGCTCATTG	206	Kurakawa et al., 2013
	GAGACTCAAGCTKRCCAGTATCAG		
*Bacteroides fragilis*	TCRGGAAGAAAGCTTGCT	162	Tong et al., 2011
	CATCCTTTACCGGAATCCT		
*Bacteroides uniformis*	TCTTCCGCATGGTAGAACTATTA	112	Tong et al., 2011
	ACCGTGTCTCAGTTCCAATGTG		
*Bacteroides fragilis* toxin	TGAAGTTAGTGCCCAGATGC	150	Zamani et al., 2017
	CAGTAAAGCCTTCCAGTCC		

### Statistical analysis

SPSS 20.0 statistical software was used for statistical analyses. Patient characteristics were compared using unpaired Student’s *t*-test, Wilcoxon rank-sum test, or χ2 test as appropriate. Student’s *t*-test was used to analyse the differential bacteria between metastatic and non-metastatic CRC tissue. ROC curve analysis was used to determine the diagnostic value of serum biomarkers or selected bacteria in patients with CRC. Other diagnostic parameters were also evaluated, including sensitivity, specificity, cut-off value, and area under the ROC curve (AUC) with 95% confidence interval (CI), to assess the discrimination power of individual or combined biomarkers. A *p*-value less than 0.05 was considered statistically significant.

## Results

### Patient characteristics

A total of 92 patients with CRC were included in the study, including 48 patients with metastatic CRC and 44 patients with non-metastatic CRC (Table 2). Statistical analysis of the basic clinical data indicated that age, gender, tumor size and location of tumor occurrence were not significantly associated with tumor metastasis. The differentiation degree was significantly related to CRC metastasis (*p* = 0.008), which is consistent with the understanding that if the tumor is less differentiated, it is more malignant and prone to metastasis (Derwinger et al., 2010). In addition, 38 of the 59 ulcerated CRC patients developed metastases, but only six of the 25 protuberant CRC patients were diagnosed with metastases (*p* = 0.001). This is because ulcerated CRC progresses deeper into the intestinal mucosa and is more likely to invade lymphatic and blood vessels, leading to CRC metastases (Bateman, 2022). Among the tumor markers alpha fetoprotein (AFP), carcinoembryonic antigen (CEA), and carbohydrate antigen 19-9 (CA19-9), the level of CEA was significantly associated with CRC metastasis.

**TABLE 2:** Clinical characteristics of 92 patients with colorectal cancer.

Parameter	Metastatic (*n* = 48)	Non-metastatic (*n* = 44)	*P*-value
Gender female (F/M)	15/33	15/29	0.772
Age (years)	64.60 ± 14.50	65.80 ± 10.40	0.655
Tumor size	11.58 ± 12.53	14.00 ± 15.57	0.413
Differentiation	–	–	0.008
Well	27	36	–
Moderate-poor	21	8	–
Proximal location	–	–	0.922
Right	12	10	–
Left	14	12	–
Rectum	22	22	–
Alpha fetoprotein	2.89 ± 1.66	2.97 ± 1.35	0.472
Carcinoembryonic antigen	104.47 ± 337.41	10.20 ± 27.91	0.012
Carbohydrate antigen 19-9	122.10 ± 340.65	62.67 ± 302.28	0.124
Macroscopic classification	–	–	0.001
Protuberant lesions	6	19	–
Ulcerated lesions	42	25	–

Data are presented as the mean ± SD.

### The α- or β-diversity of microbial community has no significant difference between metastatic and non-metastatic CRC tissue

To analyse the microbiota characteristics of tumor tissues in the metastatic and non-metastatic groups, we performed 16S rDNA amplicon high-throughput sequencing and subsequent bioinformatics analysis. The α-diversity of microbial communities is described by the Shannon and Chao indices (Ibrahim et al., 2019). The results showed that both indices of the two groups were not significantly different, indicating that the bacterial species diversity and richness were similar between metastatic and non-metastatic CRC tissues (Supplementary Figures 1A, B). Then, we applied non-metric multidimensional scaling analysis (NMDS) to analyse the β-diversity of the microbial communities. The results showed that the samples of non-metastatic group were not clustered together, and the β-diversity of two groups were not statistic significant (Supplementary Figure 1C).

### The bacterial composition is different between metastatic and non-metastatic CRC tissues

**FIGURE 1:**
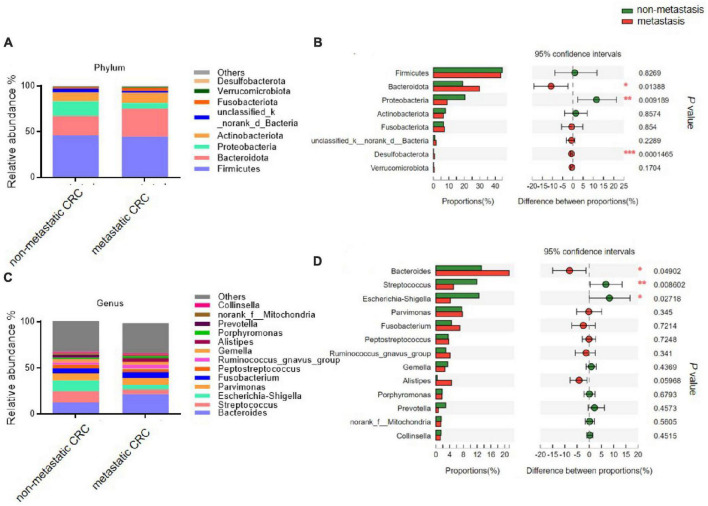
The microbial composition of tumor tissues from metastatic and non-metastatic colorectal cancer (CRC) groups. **(A)** Histograms of the predominant bacterial phyla of tumor tissues from metastatic and non-metastatic CRC groups. **(B)** The phylum-level bacterial proportion difference analysis between metastatic and non-metastatic CRC groups. **(C)** Histograms of the predominant bacterial genera of tumor tissues from metastatic and non-metastatic CRC groups. **(D)** The genus-level bacterial proportion difference analysis between metastatic and non-metastatic CRC groups. Metastatic CRC group, *n* = 48; non-metastatic CRC group, *n* = 44. The Wilcoxon rank-sum test was used in patterns **(B,D)**. *P < 0.05; **P < 0.01; ***P < 0.001.

We further analysed the composition of the microbial community from metastatic and non-metastatic CRC tissues. At the phylum level, the results showed that the intestinal bacteria in all tumor tissues were mainly from Firmicutes, Bacteroidota, Proteobacteria, Actinobacteriota, and Fusobacteriota (accounting for approximately 95%) (Figure 1A). The relative abundance of Bacteroidetes was significantly higher in the metastatic group than in the non-metastatic group (30.05 ± 21.20 vs. 18.35 ± 17.25%; *P* = 0.013), while the relative abundance of Proteobacteria was significantly lower in the metastatic group than in the non-metastatic group (9.87 ± 18.07 vs. 19.69 ± 29.13%; *P* = 0.009). In addition, Desulfobacterota, although the abundance was very low, was significantly increased in the metastatic group (0.82 ± 1.55 vs. 0.11 ± 0.35%; *P* < 0.001) (Figure 1B).

At the genus level, the bacterial composition in the tumor tissues was mainly composed of five genera: *Bacteroides*, *Streptococcus*, *Escherichia-Shigella*, *Parvimonas*, and *Fusobacterium* (Figure 1C). Among them, the relative abundance of *Bacteroides* was significantly higher in the metastatic group than in the non-metastatic group (21.67 ± 19.39 vs. 12.58 ± 12.93%; *P* = 0.049), while the relative abundances of *Streptococcus* and *Escherichia-Shigella* were significantly decreased in the metastatic group compared to the non-metastatic group (5.10 ± 11.9 vs. 23.12 ± 19.42%; *P* = 0.008 and 5.16 ± 14.65 vs. 11.66 ± 25.35%; *P* = 0.027, respectively) (Figure 1D).

The linear discriminant analysis (LDA) effect size (LEfSe; LDA score > 3.0) also found many differential bacterial species between the metastatic and non-metastatic CRC groups (Supplementary Figure 2). Interestingly, the metastatic CRC group had more relatively high abundance bacterial species than the non-metastatic CRC group, especially o_Bacteroidales, c_Bacteroidia, p_Bacteroidota, g_*Bacteroides*, f_Bacteroidaceae, g_*Alistipes*, f_Rikenellaceae, f_Oscillospiraceae, p_Desulfobacterota, and c_Desulfovibrionia, which had the highest scores, while c_Gammaproteobacteria, p_Proteobacteria, o_Enterobacterales, g_*Escherichia-Shigella*, f_Enterobacteriaceae, c_Bacilli, o_Lactobacillales, g_*Streptococcus*, f_Streptococcaceae, g_*Curvibacter*, o_Spirochaetales, and p_Spirochaetota were greatly enriched in the non-metastatic CRC group.

### *Bacteroides fragilis* and *Bacteroides uniformis* are increased in the metastatic CRC group

Bacterial composition analysis indicated that the abundance of g_*Bacteroides* was the highest among all the bacterial species and was increased in the metastatic CRC group. Then, we tried to identify the specific bacteria at the species level. In total, 37 OTUs were found to belong to g_*Bacteroides*, among which *Bacteroides fragilis* (OTU 2272), unclassified g_*Bacteroides* (OTU5969) and *Bacteroides uniformis* (OTU2249) were the three highest average abundance OTUs (Supplementary Table 1). Importantly, *Bacteroides fragilis* and *Bacteroides uniformis* showed a strong increasing trend in the metastatic CRC group compared to the non-metastatic group (*p* = 0.067 and 0.076, respectively) (Supplementary Table 1). As is reported that *Bacteroides fragilis* toxin (BFT) is the potential substance promoting tumorigenesis and metastasis (Zamani et al., 2019; Liu et al., 2020), we tested the *bft* gene frequency in non-metastatic and metastatic CRC tissue samples. The results showed that 28 out of 44 (63.6%) non-metastatic and 41 out of 48 (85.4%) metastatic CRC samples are *bft* gene positive (Supplementary Figure 3).

### The main differential bacteria are confirmed by qPCR

**FIGURE 2:**
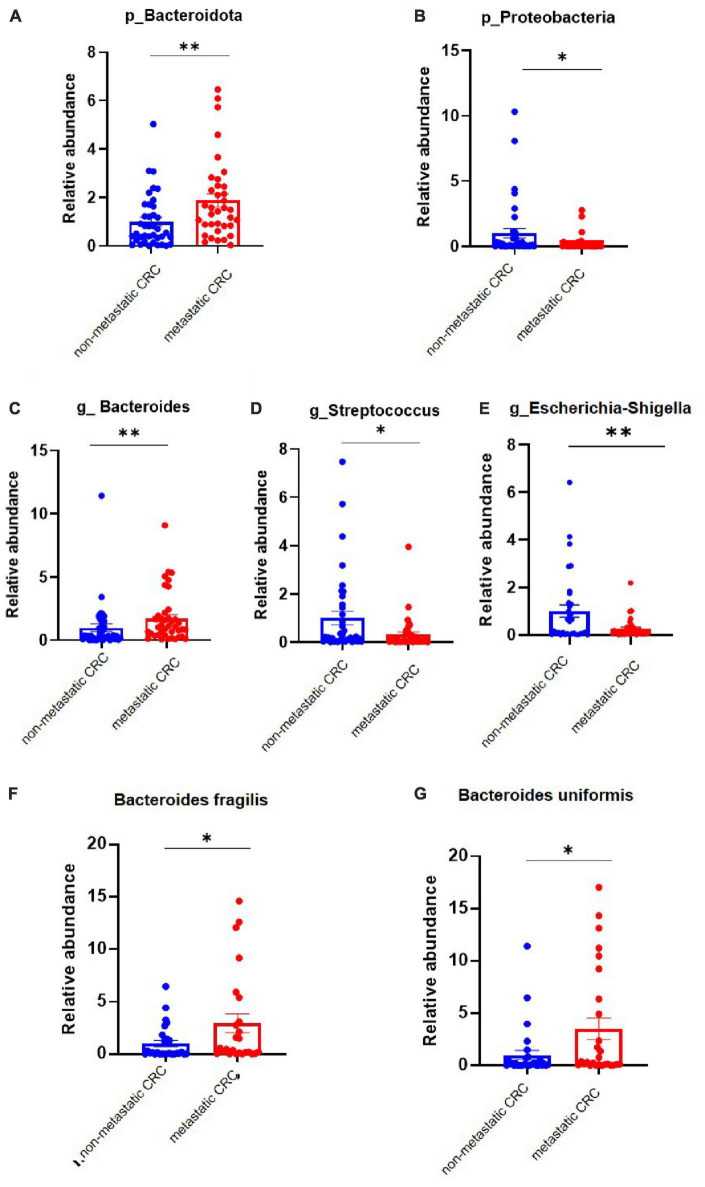
The differential bacteria between the metastatic and non-metastatic colorectal cancer (CRC) groups were confirmed by polymerase chain reaction (PCR). **(A,B)** Relative abundance of the Bacteroidetes and Proteobacteria phyla. Metastatic CRC group, *n* = 48; non-metastatic CRC group, *n* = 44. **(C–E)** Relative abundance of the *Bacteroides*, *Streptococcus*, and *Escherichia-Shigella* genera. Data below the limit of detection were removed; metastatic CRC group, *n* = 30; non-metastatic CRC group, *n* = 27. **(F,G)** Relative abundance of *Bacaeroides fragilis* and *Bacteroides uniformis*. Metastatic CRC group, *n* = 48; non-metastatic CRC group, *n* = 44. Data are presented as the mean ± SEM; *p < 0.05; **p < 0.01 by unpaired Student’s *t*-test.

To confirm the high-throughput sequencing results, we performed a qPCR experiment to quantify the main differential species in tumor tissues. The results showed that at the phylum level, the abundance of Bacteroidota was increased in metastatic CRC tissue, while that of Proteobacteria was decreased (Figures 2A, B), but the results of Desulfobacterota were lacking because its abundance was lower than the limit of detection by qPCR in this study; at the genus level, the *Bacteroides* abundance increased, but the *Streptococcus* and *Escherichia-Shigella* abundances significantly decreased in the metastatic CRC group (Figures 2C–E). In addition, we tested the abundance of *Bacteroides fragilis* and *Bacteroides uniformis*, and the results showed that they were greatly increased in metastatic CRC tissues (Figures 2F, G).

### ROC analyses of differential bacteria in diagnostic models for CRC metastasis

**FIGURE 3:**
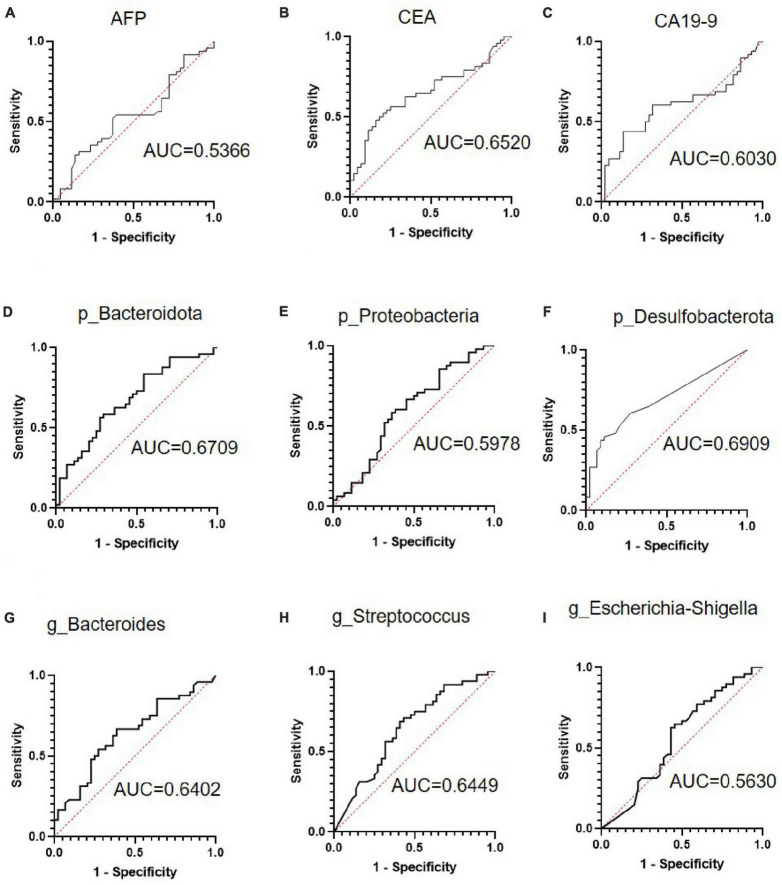
The receiver operating characteristic (ROC) curve analysis of serum tumor markers and selected bacteria in predicting the metastasis of colorectal cancer (CRC). **(A–C)** ROC curve analysis of serum tumor markers alpha fetoprotein (AFP), carcinoembryonic antigen (CEA), and carbohydrate antigen 19-9 (CA19-9) in patients with CRC. **(D–F)** ROC curve analyses of Bacteroidota, Proteobacteria, and Desulfobacterota phyla in patients with CRC. **(G–I)** ROC curve analysis of the *Bacteroides*, *Streptococcus*, and *Escherichia-Shigella* genera in patients with CRC.

First, we examined the diagnostic efficiency of the serum markers AFP, CEA, and CA19-9 in CRC metastasis. As shown in Figures 3A–C, the area under the curve (AUC) of CEA (0.652, 95% CI: 0.5387–0.7652, *p* = 0.012) was the largest, with a sensitivity of 0.479 and specificity of 0.8409 at the optimal cut-off value of 8.875. Next, we examined the diagnostic efficiency of differential bacteria in CRC metastasis. The AUCs of the ROC curves of p_Bacteroidota, p_Proteobacteria, p_Desulfobacterota, g_*Bacteroides* g_*Streptococcus*, and g*_Escherichia-Shigella* were 0.6709 (95% CI: 0.5609–0.7810, *P* = 0.0048), 0.5978 (95% CI: 0.4801–0.7155 *P* = 0.1065), 0.6906 (95% CI: 0.5822–0.7990, *P* = 0.0017), 0.6402 (95% CI: 0.5269–0.7534, *P* = 0.0207), 0.6449 (95% CI: 0.5313–0.7584, *P* = 0.0168), and 0.5630 (95% CI: 0.4430–0.6829, *P* = 0.2985), respectively (Figures 3D–I). These results indicated that the differential bacterial levels of CRC tissue groups possessed a moderate diagnostic efficiency for CRC metastasis.

**FIGURE 4:**
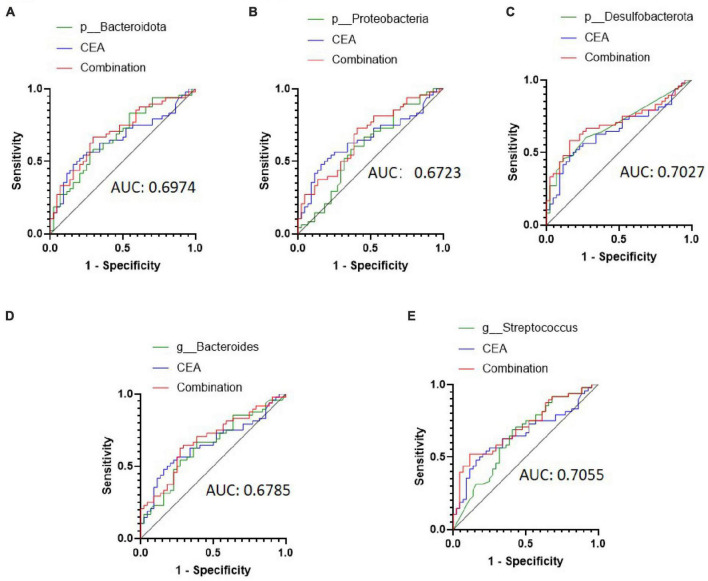
Receiver operating characteristic (ROC) curve analysis of carcinoembryonic antigen (CEA) combined with selected bacteria in predicting the metastasis of colorectal cancer (CRC). **(A–C)** ROC curve analysis of CEA combined with patients with Bacteroidota, Proteobacteria, and Desulfobacterota phyla. **(D,E)** ROC curve analysis of CEA combined with patients with *the Bacteroides* and *Streptococcus* genera.

Then, we attempted to improve the diagnostic efficacy by combining CEA with selected bacteria. The combination ROC curve of CEA with p_Bacteroidota, p_Proteobacteria, p_Desulfobacterota, g_*Bacteroides* and, or g_*Streptococcus* was drawn (Figures 4A–E), and the AUC was 0.6974 (95% CI: 0.5903–0.8046, *P* = 0.0011), 0.6723 (95% CI: 0.5628–0.7819, *P* = 0.0044), 0.7027 (95% CI: 0.5942–0.8111, *P* < 0.001), 0.6785 (95% CI: 0.5690–0.7880, *P* = 0.0032), 0.7055 (95% CI: 0.5996–0.8114, *P* < 0.001), respectively. Therefore, combination analyses obtained a higher diagnostic efficiency for CRC metastasis.

### Tumor adjacent tissues of metastatic and non-metastatic CRC show microbial composition differences

**FIGURE 5:**
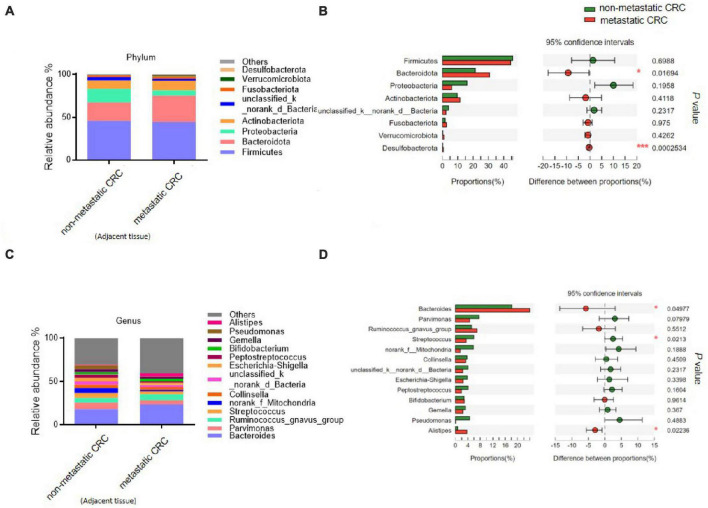
The microbial composition of tumor-adjacent tissues from metastatic and non-metastatic colorectal cancer (CRC) groups. **(A)** Histograms of the predominant bacterial phyla of tumor-adjacent tissues from metastatic and non-metastatic CRC groups. **(B)** The phylum-level bacterial proportion difference analysis of tumor-adjacent tissues from metastatic and non-metastatic CRC groups. **(C)** Histograms of the predominant bacterial genera of tumor-adjacent tissues from metastatic and non-metastatic CRC groups. **(D)** The genus-level bacterial proportion difference analysis of tumor-adjacent tissues from metastatic and non-metastatic CRC groups. Tumor-adjacent tissue of the metastatic CRC group, *n* = 48; tumor-adjacent tissue of the non-metastatic CRC group, *n* = 44. The Wilcoxon rank-sum test was used in patterns **(B,D)**. *P < 0.05; ***P < 0.001.

To explore whether the differential bacteria only existed in tumor tissue or existed in other normal intestinal tissues, we analysed the microbial composition of tumor-adjacent tissues from these patients. The results showed that at the phylum level, the relative abundances of Bacteroidota and Desulfobacterota were significantly higher in the adjacent tissue of the metastatic CRC group than in the adjacent tissue of the non-metastatic CRC group (30.78 ± 20.13 vs. 21.46 ± 20.42% and 0.56 ± 1.19 vs. 0.17 ± 0.4%, respectively) (Figures 5A, B), while the relative abundance of Proteobacteria showed a decreased trend in the adjacent tissue of the metastatic group compared to the non-metastatic group (6.02 ± 6.88 vs. 16.03 ± 25.72%) (Figures 5A, B), although there was no statistical significance. At the genus level, the relative abundance of *Bacteroides* in the metastatic group was significantly higher than that in the non-metastatic group (23.89 ± 19.31 vs. 18.16 ± 19.66%) (Figures 5C, D), while *Streptococcus* was significantly lower than that in the non-metastatic group (3.44 ± 5.6 vs. 5.9 ± 7.43%) (Figures 5C, D). These results indicated that the differential bacteria in the adjacent and tumor tissues of metastatic and non-metastatic CRC were consistent, meaning that the CRC metastasis associated bacteria are not specifically enriched in tumor tissues alone but are present in a larger area of the intestinal tract of CRC patients.

## Discussion

The harmonious intestinal microbiota, inhabiting the gut lumen, plays a crucial role in gut health (Thursby and Juge, 2017). However, in pathological situations, certain symbiotic bacteria adhere to or invade the intestinal mucosa, which can affect the progression of intestinal diseases, such as colorectal cancer (Tomkovich et al., 2019). In this study, we revealed the potential bacteria that associate with CRC metastasis, the leading cause of CRC death, by systematically analysing the characteristics of the tissue-associated microbiota collected from the non-metastatic and metastatic CRC groups.

We collected mucosal tissues from CRC patients who underwent surgical operation and extracted the DNA of tissue-associated bacteria. The function of tissue-associated bacteria in CRC progression may differ from that of luminal bacteria (Durbán et al., 2011; Chen, 2018). The gut microbiota in the lumen usually indirectly affects epithelial cells, such as by metabolites (Dalal et al., 2021), but tissue-associated bacteria are believed to stimulate intestinal cells directly and intensely (Chen et al., 2012). Therefore, mucosal bacteria should play more important roles than the gut microbiota in CRC progression. In addition, we found that all the tissues from CRC patients contained more mucosal bacteria than those from healthy individuals (data not shown). The probable reason is that the colon of CRC patients is associated with a reduced intestinal barrier (Sun et al., 2022).

Our study found that the composition of the flora of the two groups showed a great difference. We noted that *Bacteroides* was the most abundant bacterium for tissue adhesion and was significantly enriched in the metastatic group. *Bacteroides fragilis* and *Bacteroides uniformi*s were two species that were significantly elevated in the metastatic group. In fact, it was reported that *Bacteroides fragilis* was higher in the stool of CRC patients than in healthy individuals. In addition, *Bacteroides fragilis* has the ability to penetrate the colonic mucus and resides deep within crypt channels (Lee et al., 2013). Thus, its abundance was very high in CRC intestinal tissue (Li S. et al., 2021). Mechanistically, *Bacteroides fragilis* can secrete *B. fragilis* toxin and induce stemness in CRC by upregulating Jumonji domain-containing protein 2B (JMJD2B) levels in a TLR4-NFAT5-dependent pathway (Liu et al., 2020). Recently, Parida et al. (2021) found that enterotoxigenic *Bacteroides fragilis* (ETBF) is present in breast tumor tissue, triggers epithelial hyperplasia and augments breast cancer growth and metastasis via the β-catenin and Notch1 pathways. Our results indicated that the abundance of *Bacteroides fragilis* increased significantly, and *bft* gene was more prevalent in metastatic CRC samples, which is consistent with its role in bowel cancer progression and metastasis. *Bacteroides uniformis* is usually known as a harmless bacterium, but some other studies and our study identified that its abundance increased in the CRC group. Further studies are needed to clarify the potential tumor-promoting function of *Bacteroides uniformis*.

The abundance of *Streptococcus* was decreased in the metastatic CRC group. Many studies have reported that different species of *Streptococcus* play different roles in CRC. Some species, such as *Streptococcus gallolyticus*, strongly associated with the occurrence of colorectal cancer are known as tumor-promoting bacteria (Aymeric et al., 2018). Nevertheless, Li Q. et al. (2021) reported that *Streptococcus thermophiles*, which is depleted in stool samples of patients with CRC, plays a tumor-suppressive role by secreting β-galactosidase to maintain high galactose content throughout the gastrointestinal tract and then inhibit the Hippo pathway in tumour tissues. In our study, *Streptococcus* may act as a tumor-inhibiting bacterium by an unknown mechanism. Our ongoing work will try to identify specific species and research their antitumor functions.

In addition, our results showed that the main differential bacteria of tumor-adjacent tissue are similar to those of tumor tissues, indicating that in CRC patients, tissue-associated bacteria may be present in a wider range of intestinal tissues rather than only in the tumor. Similarly, Boleij et al. (2015) reported that the *bft* gene, which plays an important role in the pathogenesis of human CRC, is not limited to tumors but spans a larger portion of the colonic mucosa. Therefore, further study is needed to comprehensively evaluate the impact of CRC metastasis associated bacteria on intestinal health.

## Data availability statement

The datasets presented in this study can be found in online repositories and accession number can be found below: https://www.ncbi.nlm.nih.gov/, PRJNA916596.

## Ethics statement

The studies involving human participants were reviewed and approved by the Ethical Committee of the Affiliated Hospital of Medical School, Ningbo University (KS202111002). The patients/participants provided their written informed consent to participate in this study. Written informed consent was obtained from the individual(s) for the publication of any potentially identifiable images or data included in this article.

## Author contributions

DS and YZ conceived and designed the project. PZ, ZD, and TL recruited patients and collected tissue samples. PZ performed qPCR experiments. PZ, ZD, YX, ZX, YH, and DS analysed the data. PZ and DS prepared the manuscript. DS and YZ wrote and reviewed the final version of the text. All authors contributed to finalizing the manuscript, read, and approved the manuscript.

## Footnotes

1
http://qiime.org/install/index.html

2
http://drive5.com/uparse/

3
http://huttenhower.sph.harvard.edu/LEfSe
