# Supplementary material for: Differences in tissue-associated bacteria between metastatic and non-metastatic colorectal cancer
Source: Front Microbiol. 2023 Jun 9;14:1133607. doi: 10.3389/fmicb.2023.1133607 (PMC10289161; doi:10.3389/fmicb.2023.1133607)
Supplement: Supplementary file 1 [file Presentation_1.PPTX]

## Slide 1
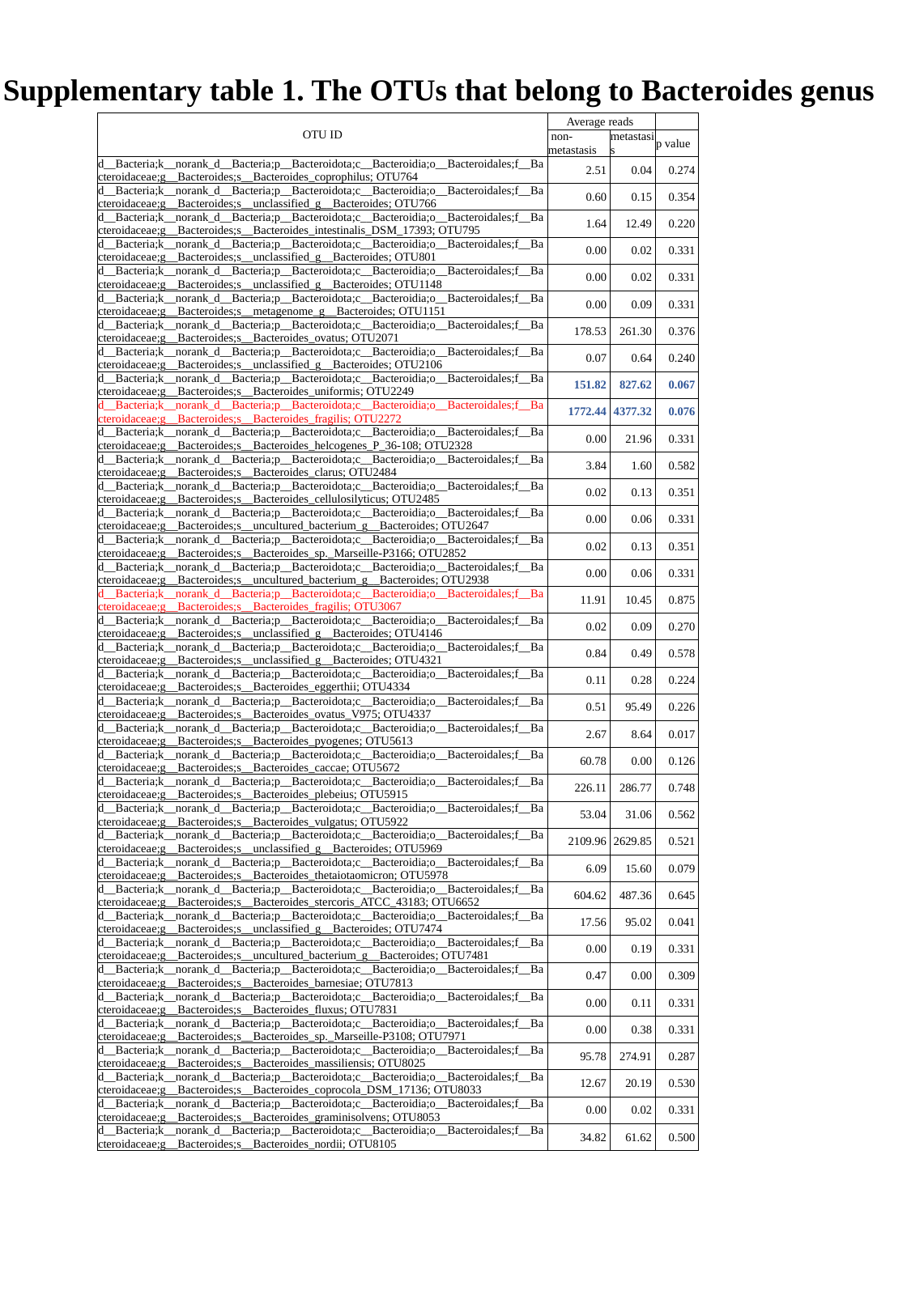

Supplementary table 1. The OTUs that belong to Bacteroides genus
| OTU ID | Average reads | | |
| --- | --- | --- | --- |
| | non-metastasis | metastasis | p value |
| d\_\_Bacteria;k\_\_norank\_d\_\_Bacteria;p\_\_Bacteroidota;c\_\_Bacteroidia;o\_\_Bacteroidales;f\_\_Bacteroidaceae;g\_\_Bacteroides;s\_\_Bacteroides\_coprophilus; OTU764 | 2.51 | 0.04 | 0.274 |
| d\_\_Bacteria;k\_\_norank\_d\_\_Bacteria;p\_\_Bacteroidota;c\_\_Bacteroidia;o\_\_Bacteroidales;f\_\_Bacteroidaceae;g\_\_Bacteroides;s\_\_unclassified\_g\_\_Bacteroides; OTU766 | 0.60 | 0.15 | 0.354 |
| d\_\_Bacteria;k\_\_norank\_d\_\_Bacteria;p\_\_Bacteroidota;c\_\_Bacteroidia;o\_\_Bacteroidales;f\_\_Bacteroidaceae;g\_\_Bacteroides;s\_\_Bacteroides\_intestinalis\_DSM\_17393; OTU795 | 1.64 | 12.49 | 0.220 |
| d\_\_Bacteria;k\_\_norank\_d\_\_Bacteria;p\_\_Bacteroidota;c\_\_Bacteroidia;o\_\_Bacteroidales;f\_\_Bacteroidaceae;g\_\_Bacteroides;s\_\_unclassified\_g\_\_Bacteroides; OTU801 | 0.00 | 0.02 | 0.331 |
| d\_\_Bacteria;k\_\_norank\_d\_\_Bacteria;p\_\_Bacteroidota;c\_\_Bacteroidia;o\_\_Bacteroidales;f\_\_Bacteroidaceae;g\_\_Bacteroides;s\_\_unclassified\_g\_\_Bacteroides; OTU1148 | 0.00 | 0.02 | 0.331 |
| d\_\_Bacteria;k\_\_norank\_d\_\_Bacteria;p\_\_Bacteroidota;c\_\_Bacteroidia;o\_\_Bacteroidales;f\_\_Bacteroidaceae;g\_\_Bacteroides;s\_\_metagenome\_g\_\_Bacteroides; OTU1151 | 0.00 | 0.09 | 0.331 |
| d\_\_Bacteria;k\_\_norank\_d\_\_Bacteria;p\_\_Bacteroidota;c\_\_Bacteroidia;o\_\_Bacteroidales;f\_\_Bacteroidaceae;g\_\_Bacteroides;s\_\_Bacteroides\_ovatus; OTU2071 | 178.53 | 261.30 | 0.376 |
| d\_\_Bacteria;k\_\_norank\_d\_\_Bacteria;p\_\_Bacteroidota;c\_\_Bacteroidia;o\_\_Bacteroidales;f\_\_Bacteroidaceae;g\_\_Bacteroides;s\_\_unclassified\_g\_\_Bacteroides; OTU2106 | 0.07 | 0.64 | 0.240 |
| d\_\_Bacteria;k\_\_norank\_d\_\_Bacteria;p\_\_Bacteroidota;c\_\_Bacteroidia;o\_\_Bacteroidales;f\_\_Bacteroidaceae;g\_\_Bacteroides;s\_\_Bacteroides\_uniformis; OTU2249 | 151.82 | 827.62 | 0.067 |
| d\_\_Bacteria;k\_\_norank\_d\_\_Bacteria;p\_\_Bacteroidota;c\_\_Bacteroidia;o\_\_Bacteroidales;f\_\_Bacteroidaceae;g\_\_Bacteroides;s\_\_Bacteroides\_fragilis; OTU2272 | 1772.44 | 4377.32 | 0.076 |
| d\_\_Bacteria;k\_\_norank\_d\_\_Bacteria;p\_\_Bacteroidota;c\_\_Bacteroidia;o\_\_Bacteroidales;f\_\_Bacteroidaceae;g\_\_Bacteroides;s\_\_Bacteroides\_helcogenes\_P\_36-108; OTU2328 | 0.00 | 21.96 | 0.331 |
| d\_\_Bacteria;k\_\_norank\_d\_\_Bacteria;p\_\_Bacteroidota;c\_\_Bacteroidia;o\_\_Bacteroidales;f\_\_Bacteroidaceae;g\_\_Bacteroides;s\_\_Bacteroides\_clarus; OTU2484 | 3.84 | 1.60 | 0.582 |
| d\_\_Bacteria;k\_\_norank\_d\_\_Bacteria;p\_\_Bacteroidota;c\_\_Bacteroidia;o\_\_Bacteroidales;f\_\_Bacteroidaceae;g\_\_Bacteroides;s\_\_Bacteroides\_cellulosilyticus; OTU2485 | 0.02 | 0.13 | 0.351 |
| d\_\_Bacteria;k\_\_norank\_d\_\_Bacteria;p\_\_Bacteroidota;c\_\_Bacteroidia;o\_\_Bacteroidales;f\_\_Bacteroidaceae;g\_\_Bacteroides;s\_\_uncultured\_bacterium\_g\_\_Bacteroides; OTU2647 | 0.00 | 0.06 | 0.331 |
| d\_\_Bacteria;k\_\_norank\_d\_\_Bacteria;p\_\_Bacteroidota;c\_\_Bacteroidia;o\_\_Bacteroidales;f\_\_Bacteroidaceae;g\_\_Bacteroides;s\_\_Bacteroides\_sp.\_Marseille-P3166; OTU2852 | 0.02 | 0.13 | 0.351 |
| d\_\_Bacteria;k\_\_norank\_d\_\_Bacteria;p\_\_Bacteroidota;c\_\_Bacteroidia;o\_\_Bacteroidales;f\_\_Bacteroidaceae;g\_\_Bacteroides;s\_\_uncultured\_bacterium\_g\_\_Bacteroides; OTU2938 | 0.00 | 0.06 | 0.331 |
| d\_\_Bacteria;k\_\_norank\_d\_\_Bacteria;p\_\_Bacteroidota;c\_\_Bacteroidia;o\_\_Bacteroidales;f\_\_Bacteroidaceae;g\_\_Bacteroides;s\_\_Bacteroides\_fragilis; OTU3067 | 11.91 | 10.45 | 0.875 |
| d\_\_Bacteria;k\_\_norank\_d\_\_Bacteria;p\_\_Bacteroidota;c\_\_Bacteroidia;o\_\_Bacteroidales;f\_\_Bacteroidaceae;g\_\_Bacteroides;s\_\_unclassified\_g\_\_Bacteroides; OTU4146 | 0.02 | 0.09 | 0.270 |
| d\_\_Bacteria;k\_\_norank\_d\_\_Bacteria;p\_\_Bacteroidota;c\_\_Bacteroidia;o\_\_Bacteroidales;f\_\_Bacteroidaceae;g\_\_Bacteroides;s\_\_unclassified\_g\_\_Bacteroides; OTU4321 | 0.84 | 0.49 | 0.578 |
| d\_\_Bacteria;k\_\_norank\_d\_\_Bacteria;p\_\_Bacteroidota;c\_\_Bacteroidia;o\_\_Bacteroidales;f\_\_Bacteroidaceae;g\_\_Bacteroides;s\_\_Bacteroides\_eggerthii; OTU4334 | 0.11 | 0.28 | 0.224 |
| d\_\_Bacteria;k\_\_norank\_d\_\_Bacteria;p\_\_Bacteroidota;c\_\_Bacteroidia;o\_\_Bacteroidales;f\_\_Bacteroidaceae;g\_\_Bacteroides;s\_\_Bacteroides\_ovatus\_V975; OTU4337 | 0.51 | 95.49 | 0.226 |
| d\_\_Bacteria;k\_\_norank\_d\_\_Bacteria;p\_\_Bacteroidota;c\_\_Bacteroidia;o\_\_Bacteroidales;f\_\_Bacteroidaceae;g\_\_Bacteroides;s\_\_Bacteroides\_pyogenes; OTU5613 | 2.67 | 8.64 | 0.017 |
| d\_\_Bacteria;k\_\_norank\_d\_\_Bacteria;p\_\_Bacteroidota;c\_\_Bacteroidia;o\_\_Bacteroidales;f\_\_Bacteroidaceae;g\_\_Bacteroides;s\_\_Bacteroides\_caccae; OTU5672 | 60.78 | 0.00 | 0.126 |
| d\_\_Bacteria;k\_\_norank\_d\_\_Bacteria;p\_\_Bacteroidota;c\_\_Bacteroidia;o\_\_Bacteroidales;f\_\_Bacteroidaceae;g\_\_Bacteroides;s\_\_Bacteroides\_plebeius; OTU5915 | 226.11 | 286.77 | 0.748 |
| d\_\_Bacteria;k\_\_norank\_d\_\_Bacteria;p\_\_Bacteroidota;c\_\_Bacteroidia;o\_\_Bacteroidales;f\_\_Bacteroidaceae;g\_\_Bacteroides;s\_\_Bacteroides\_vulgatus; OTU5922 | 53.04 | 31.06 | 0.562 |
| d\_\_Bacteria;k\_\_norank\_d\_\_Bacteria;p\_\_Bacteroidota;c\_\_Bacteroidia;o\_\_Bacteroidales;f\_\_Bacteroidaceae;g\_\_Bacteroides;s\_\_unclassified\_g\_\_Bacteroides; OTU5969 | 2109.96 | 2629.85 | 0.521 |
| d\_\_Bacteria;k\_\_norank\_d\_\_Bacteria;p\_\_Bacteroidota;c\_\_Bacteroidia;o\_\_Bacteroidales;f\_\_Bacteroidaceae;g\_\_Bacteroides;s\_\_Bacteroides\_thetaiotaomicron; OTU5978 | 6.09 | 15.60 | 0.079 |
| d\_\_Bacteria;k\_\_norank\_d\_\_Bacteria;p\_\_Bacteroidota;c\_\_Bacteroidia;o\_\_Bacteroidales;f\_\_Bacteroidaceae;g\_\_Bacteroides;s\_\_Bacteroides\_stercoris\_ATCC\_43183; OTU6652 | 604.62 | 487.36 | 0.645 |
| d\_\_Bacteria;k\_\_norank\_d\_\_Bacteria;p\_\_Bacteroidota;c\_\_Bacteroidia;o\_\_Bacteroidales;f\_\_Bacteroidaceae;g\_\_Bacteroides;s\_\_unclassified\_g\_\_Bacteroides; OTU7474 | 17.56 | 95.02 | 0.041 |
| d\_\_Bacteria;k\_\_norank\_d\_\_Bacteria;p\_\_Bacteroidota;c\_\_Bacteroidia;o\_\_Bacteroidales;f\_\_Bacteroidaceae;g\_\_Bacteroides;s\_\_uncultured\_bacterium\_g\_\_Bacteroides; OTU7481 | 0.00 | 0.19 | 0.331 |
| d\_\_Bacteria;k\_\_norank\_d\_\_Bacteria;p\_\_Bacteroidota;c\_\_Bacteroidia;o\_\_Bacteroidales;f\_\_Bacteroidaceae;g\_\_Bacteroides;s\_\_Bacteroides\_barnesiae; OTU7813 | 0.47 | 0.00 | 0.309 |
| d\_\_Bacteria;k\_\_norank\_d\_\_Bacteria;p\_\_Bacteroidota;c\_\_Bacteroidia;o\_\_Bacteroidales;f\_\_Bacteroidaceae;g\_\_Bacteroides;s\_\_Bacteroides\_fluxus; OTU7831 | 0.00 | 0.11 | 0.331 |
| d\_\_Bacteria;k\_\_norank\_d\_\_Bacteria;p\_\_Bacteroidota;c\_\_Bacteroidia;o\_\_Bacteroidales;f\_\_Bacteroidaceae;g\_\_Bacteroides;s\_\_Bacteroides\_sp.\_Marseille-P3108; OTU7971 | 0.00 | 0.38 | 0.331 |
| d\_\_Bacteria;k\_\_norank\_d\_\_Bacteria;p\_\_Bacteroidota;c\_\_Bacteroidia;o\_\_Bacteroidales;f\_\_Bacteroidaceae;g\_\_Bacteroides;s\_\_Bacteroides\_massiliensis; OTU8025 | 95.78 | 274.91 | 0.287 |
| d\_\_Bacteria;k\_\_norank\_d\_\_Bacteria;p\_\_Bacteroidota;c\_\_Bacteroidia;o\_\_Bacteroidales;f\_\_Bacteroidaceae;g\_\_Bacteroides;s\_\_Bacteroides\_coprocola\_DSM\_17136; OTU8033 | 12.67 | 20.19 | 0.530 |
| d\_\_Bacteria;k\_\_norank\_d\_\_Bacteria;p\_\_Bacteroidota;c\_\_Bacteroidia;o\_\_Bacteroidales;f\_\_Bacteroidaceae;g\_\_Bacteroides;s\_\_Bacteroides\_graminisolvens; OTU8053 | 0.00 | 0.02 | 0.331 |
| d\_\_Bacteria;k\_\_norank\_d\_\_Bacteria;p\_\_Bacteroidota;c\_\_Bacteroidia;o\_\_Bacteroidales;f\_\_Bacteroidaceae;g\_\_Bacteroides;s\_\_Bacteroides\_nordii; OTU8105 | 34.82 | 61.62 | 0.500 |

## Slide 2
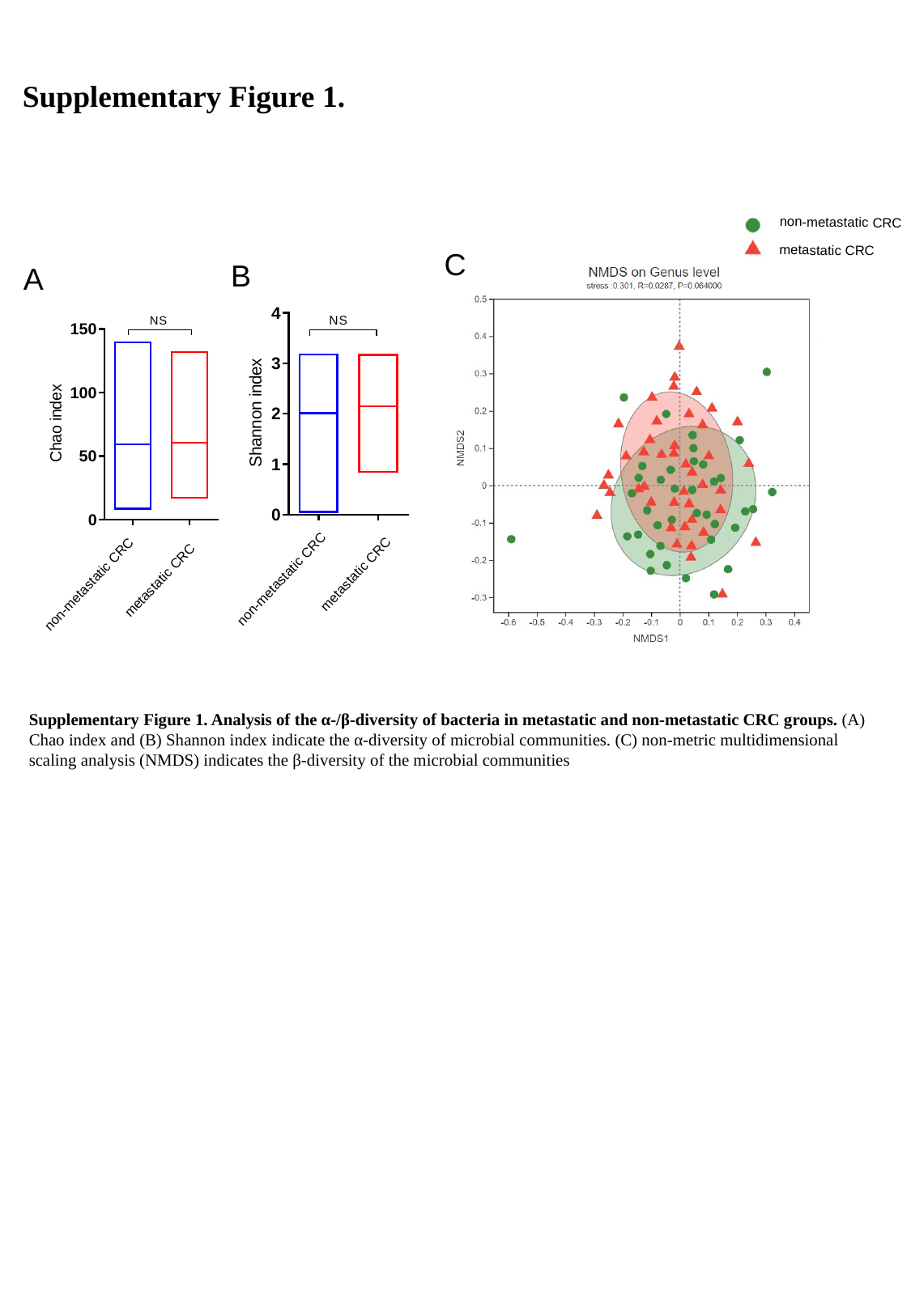

Supplementary Figure 1.
non-metastatic CRC
metastatic CRC
C
B
A
metastatic CRC
non-metastatic CRC
metastatic CRC
non-metastatic CRC
Supplementary Figure 1. Analysis of the α-/β-diversity of bacteria in metastatic and non-metastatic CRC groups. (A) Chao index and (B) Shannon index indicate the α-diversity of microbial communities. (C) non-metric multidimensional scaling analysis (NMDS) indicates the β-diversity of the microbial communities

## Slide 3
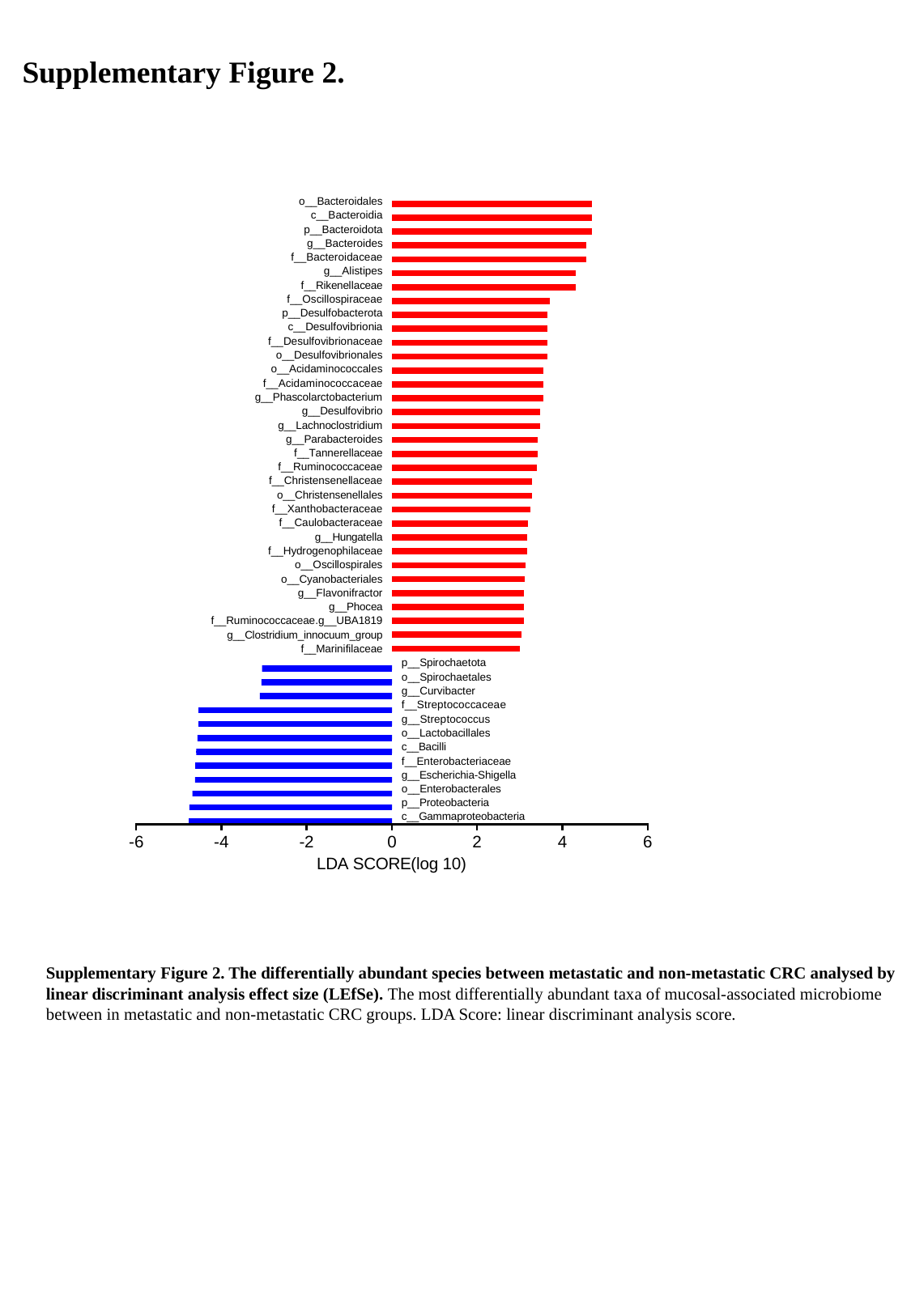

Supplementary Figure 2.
Supplementary Figure 2. The differentially abundant species between metastatic and non-metastatic CRC analysed by linear discriminant analysis effect size (LEfSe). The most differentially abundant taxa of mucosal-associated microbiome between in metastatic and non-metastatic CRC groups. LDA Score: linear discriminant analysis score.

## Slide 4
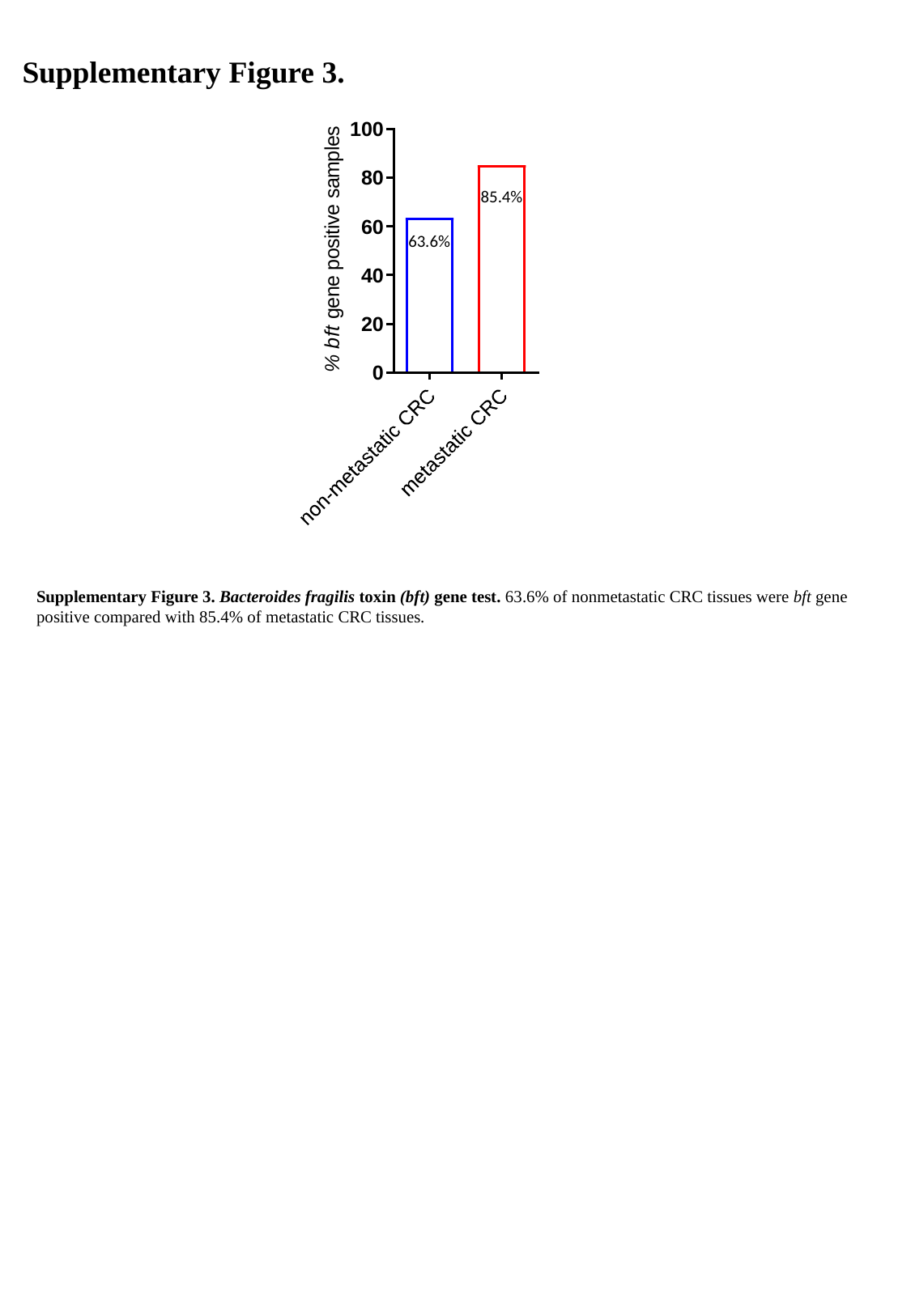

Supplementary Figure 3.
85.4%
63.6%
Supplementary Figure 3. Bacteroides fragilis toxin (bft) gene test. 63.6% of nonmetastatic CRC tissues were bft gene positive compared with 85.4% of metastatic CRC tissues.
